# Comparison of Intraoral and Extraoral Digital Scanners: Evaluation of Surface Topography and Precision

**DOI:** 10.3390/dj8020052

**Published:** 2020-05-20

**Authors:** Sang J. Lee, Soo-Woo Kim, Joshua J. Lee, Chan W. Cheong

**Affiliations:** 1 Department of Restorative Dentistry and Biomaterials Sciences, Harvard School of Dental Medicine, Boston, MA 02115, USA; joshjjlee@gmail.com (J.J.L.); Chan_cheong@hsdm.harvard.edu (C.W.C.); 2 Department of Oral Medicine, Infection, and Immunity, Harvard School of Dental Medicine, Boston, MA 02115, USA; soo-woo_kim@hsdm.harvard.edu

**Keywords:** digital dentistry, digital impression, surface topography, precision, accuracy

## Abstract

The aim of this study was to evaluate the surface topography and the precision measurements of different intraoral and extraoral digital scanners. A reference model of a maxillary arch with four implant analogs was prepared and scanned by three intraoral and two extraoral scanners. The reference model was scanned fifteen times with each digital scanning system, investigating the surface topography and precision measurements for the same-arch and cross-arch measurements. The data was exported to 3D inspection and mesh-processing software (GOM Inspect, Braunschweig, Germany). Statistical analysis was performed using a one-way Analysis of Variance (ANOVA) with the Tukey method for pairwise comparisons. The effect of parameters on generating the surface topography was analyzed by Univariate Linear Regression Analysis. Of the scanner systems evaluated, iTero (IT) exhibited the most number of triangulation points, followed by Trios 3 Shape (TR) and Straumann Cares (SC). There were no significant differences observed in the surface topography when comparing flat and contoured surfaces, the anterior and posterior position, and interproximal areas. For the precision measurement in the same quadrant, no statistical difference was noted between intra- and extraoral scanners. However, the extraoral scanners showed substantially higher precision measurements for the cross-arch measurement. Surface topography did not correlate to precision. Rather, precision correlated with the scanning mechanism. For a quadrant scanning, both intraoral and extraoral scanners are recommended, but extraoral scanners are recommended for a full-arch scanning.

## 1. Introduction

Making conventional impressions and fabricating gypsum models involve clinical and laboratory procedures that may result in cumulative errors. These processing errors are largely inherent to the handling and properties of dental materials. The conventional approaches of a physical impression made with an elastomeric impression material may affect the treatment outcomes, including accuracy, efficiency, and patients’ comfort level [1]. Despite these limitations, the conventional impression technique has been considered the gold standard in dentistry mainly due to cost-effectiveness [2].

The use of digital technology in dentistry has advanced rapidly in the past few decades. Developed in the 1950s, current CAD/CAM (computer-aided design and computer-aided manufacturing) technology has not only brought a paradigm shift in treatment workflows but also enhanced patient care. The technology has supported modeling, design, and fabrication of dental models and restorations [3]. Technological advancement in 3D imaging and the application of CAD/CAM has improved efficiency and predictability in both the surgical and restorative phases, including the dental laboratory processes [4,5,6].

Recent studies have suggested that the digital workflow improved treatment modality and that integration of digital technology simplified surgical and restorative treatment, leading to more efficient and predictable treatment outcomes. Digital workflow also allows for innovative treatment approaches, by reducing the processing errors throughout the making of conventional impressions and handling of gypsum materials [7,8,9,10]. The improvement is largely based on the advancement of digital technology in dentistry, including digital dental scanners, 3D imaging, software, and 3D object producing units.

The digital workflow itself consists of three different phases: (1) a data acquisition phase, (2) design phase, and (3) manufacturing phase [11]. During the data acquisition phase, digital scanners measure the surface points with certain accuracy and precision and define the surface topography [12]. After the data acquisition phase, the proprietary file containing the digital scanning is converted into a universal file (STL, Standard Tessellation Language). The transformation of the file takes place across different platforms independent of the initial underlying system. The scanners produce datasets with varying quality based on different scanning technologies and software processing algorithms. The quality of the scanners is evaluated in terms of accuracy. The accuracy is generally described in two ways: trueness and precision [13,14]. Trueness refers to the closeness of a measurement to the actual value and precision expresses the degree of reproducibility between repeated measurements. The surface topography produced from the scanners defines the nature of a surface and is often used to describe the surface characteristics, such as surface roughness, waviness, and flatness [15].

Typically, three types of scanners are used: mechanical scanners with a touch-probe, laser scanners, or white light scanners [16,17,18]. A few manufacturers have reported the accuracy and precision value of their scanners. However, their clinical significance has still remained in question, mainly due to the fact that testing parameters are not clearly defined. There are many published studies comparing the accuracy of various digital scanners and different types of restorations such as partial and complete arches [9,13,14]. However, a few rigorous studies exist in the literature in terms of the surface topography by the different anatomical areas of the teeth and its correlation to precision.

Accurate and precise digitization is a crucial step in the digital workflow, and the clinical viability of digital impression systems should be validated in the data acquisition and digitization step. The objective of this study is to evaluate the surface topography and precision when digitizing the various anatomical areas and the different types of arch scans using different dental scanners. The null hypothesis is that there is no statistical difference between the surface topography and precision in terms of anatomical areas and the different types of dental scanners.

## 2. Materials and Methods 

### 2.1. Digital Scanners

**Figure 1:**
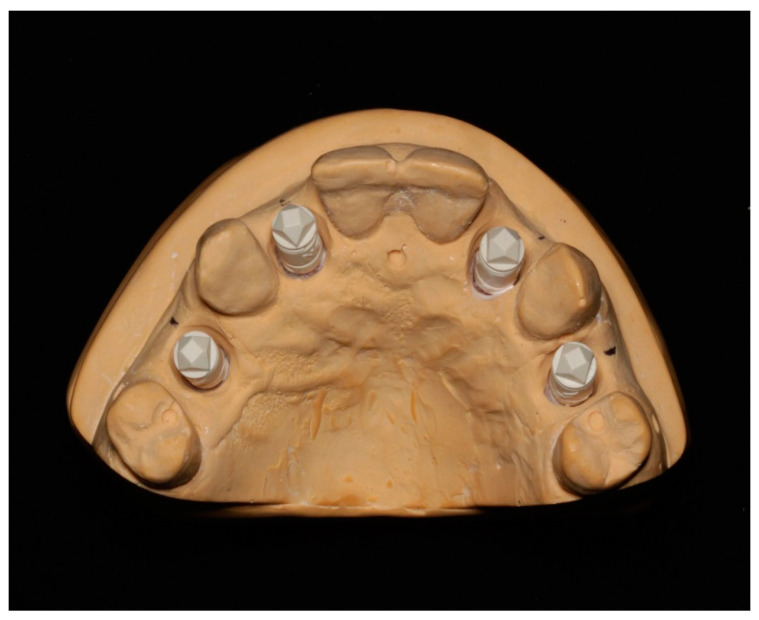
A Reference Master model.

The reference model of a maxillary arch (Figure 1) was prepared with four implant analogs (Straumann, Basel, Switzerland) at the location of the maxillary lateral incisors and first premolars (#5, 7, 10 and 12, respectively by Universal Numbering System) by customizing a typodont (Columbia Dentoform, Lancaster, PA, USA). The reference model was fabricated from a digitally scannable type IV gypsum material compatible with optical scanning devices (GC Fujirock EP OptiXscan, Tokyo, Japan) and scanned by intraoral and extraoral scanning systems underbalanced lighting in a dental clinic (average temperature 5500K and color rendering index >90). The light setting stayed consistent throughout the scanning process. This study used three intraoral scanning systems—CEREC Omnicam (CO) (Dentsply Sirona York, PA, USA), iTero HD2.9 (IT) (Align Technology, San Jose, CA, USA), TRIOS 3Shape 3 Basic (TR) (3Shape, Copenhagen, Denmark), and two extraoral scanners—3Shape D700 (TS) (3Shape, Copenhagen, Denmark) and Straumann Cares 3 series (SC) (Straumann, Basel, Switzerland). The specifications of the scanners are summarized in Table 1.

### 2.2. Data Collection 

A Straumann scanbody (Straumann, Basel, Switzerland) was placed onto each implant analog in the reference model. The positions of implant scanbodies on the maxillary lateral incisors and first premolars (#5, 7, 10, and 12) were identified in each scanner’s proprietary software for model configuration, according to the respective digital impression systems. Each scanbody consists of a top-flap rectangle with four sloping pentagons bordering the top rectangle. The reference model was scanned fifteen times with each digital scanning system (Table 1) by a single experienced clinician to avoid operator-biased results. None of the scanners required the application of titanium dioxide powder. Then, the digitized data was exported into an STL file format via each scanners’ respective proprietary conversion software. The digitized virtual models were inspected to confirm the complete scan of the reference model and the absence of void and missing areas. Subsequently, the data was exported to 3D inspection and mesh-processing software (GOM Inspect, Branunschweig, Germany) by which the measurement and computation of the digitized model were investigated.

### 2.3. Surface Topography Measurement on Implant Scanbody

**Figure 2:**
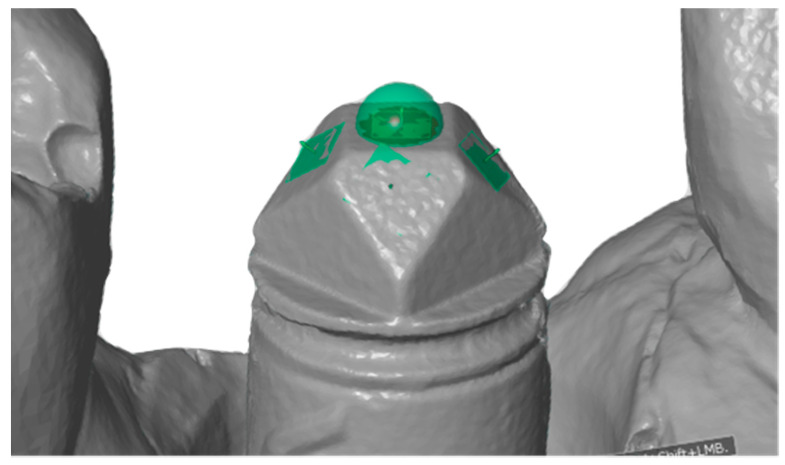
Surface topography measurement on the top surface on the scanbody.

The surface topography was evaluated by measuring computed triangulation points on a scanbody in the #5 implant position by 3D inspection and mesh-processing software (GOM Inspect, Branunschweig, Germany). The surface topography was measured by the number of triangulation points on the 3D surface image of the STL file within a defined boundary of a 0.5 mm radius sphere in the center of the top surface in the scanbody (Figure 2). Triangulation points enclosed within the circle on the surface of the scanbody were then counted. 

### 2.4. Surface Topography Measurements on Tooth Surfaces

**Figure 3:**
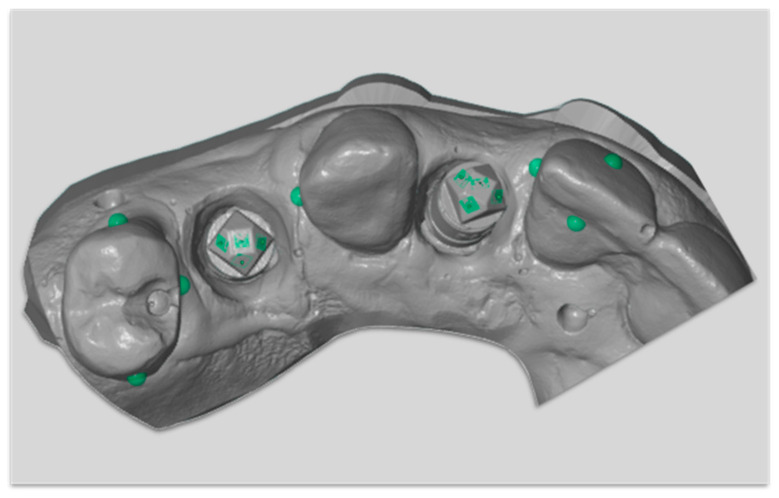
Surface topography measurement on different tooth surfaces.

The surface topography of tooth surfaces was also measured by calculating triangulation points enclosed within a sphere with 0.5 mm on the various tooth surfaces. The measured points were #4 (buccal, lingual, and mesial), #6 (distal), and #9 (buccal, lingual, and distal) as in Figure 3. The effect and relationship between the parameters were investigated. 

### 2.5. Precision Measurement between Implant Scanbodies

**Figure 4:**
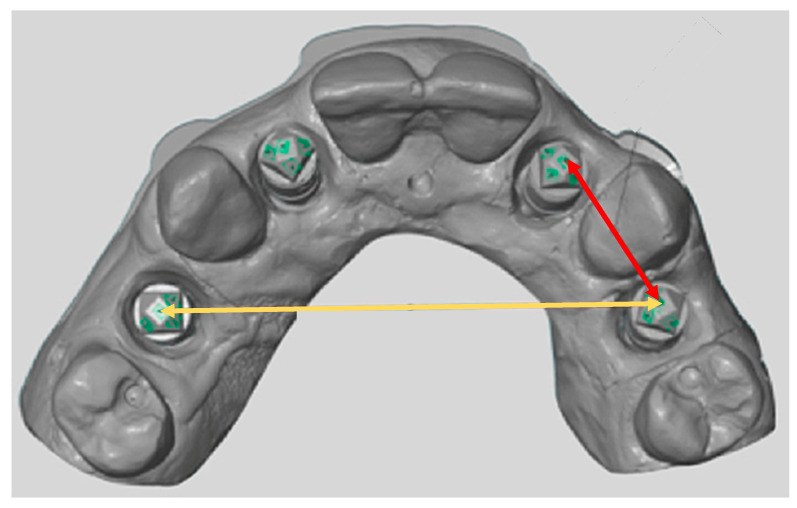
Linear measurements for cross-arch and same-quadrant measurements. Yellow arrow: cross-arch linear measurement between #5 scanbody and #12 scanbody; Red arrow: same-quadrant linear measurement between #10 implant and #12 implant.

The precision of the digital impression systems was evaluated by the linear measurements of the distance between the #10 and #12 scanbodies for the same quadrant, and #5 and #12 scanbodies for cross-arch measurement. Reproducible reference points were located from scanbodies of #5, #10, and #12. The distance between the reference points from scanbodies #10 and #12 was measured for same quadrant measurements, and the distance between the reference points from scanbodies #5 and #12 was measured for cross-arch linear measurements (Figure 4). Then, precision, by definition, is calculated by an “inverse of the square of variance (1/σ^2^)” from the measurements. 

### 2.6. Statistical Analysis

The statistical analysis was performed using a commercially available software program (SPSSs for Windows version 19.0, IBM Corp., Somers, NY, USA). Means and standard deviations were calculated for all quantitative data. For comparison of surface topography by the number of the triangulation points among different scanners, collected data of each group were compared using one-way Analysis of Variance (ANOVA) with the Tukey method for pairwise comparisons. The effect of parameters from the surface topography of the various tooth surfaces was analyzed by Univariate Linear Regression Analysis. The best-fit model was chosen by adding parameters individually to analyze until statistical significance did not change. *p* < 0.05 was considered to be statistically significant.

## 3. Results

**Figure 5:**
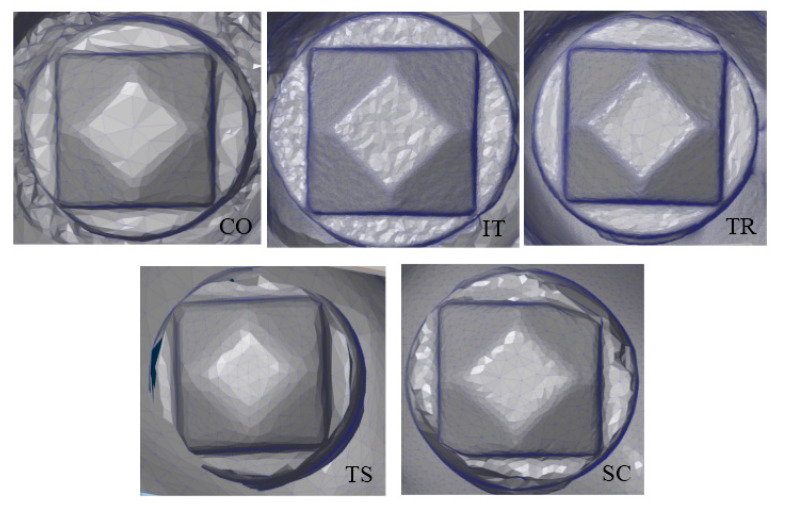
Surface topography on the top surface area of the scanbody.

**Figure 6:**
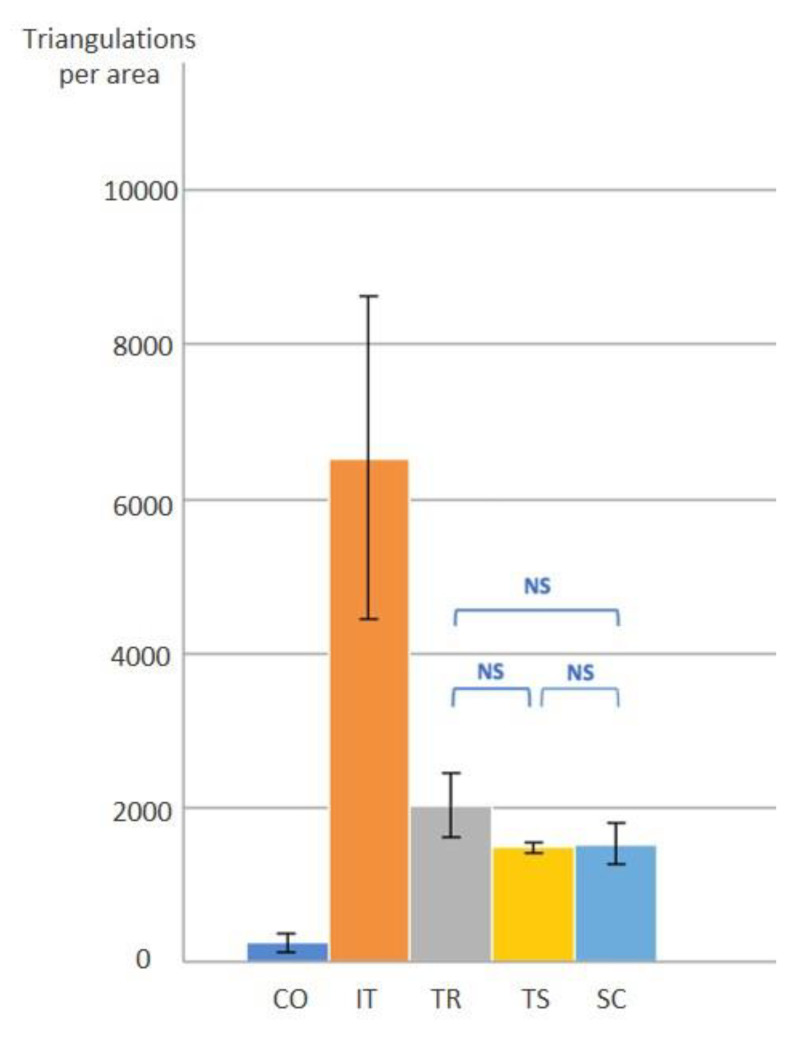
Triangulation points on the top surface area of the scanbody #5. NS: *p* <0.05.

The digitized surface of the scanbody on implant #5 is shown in Figure 5. The IT scanner had the greatest number of triangulation points with an average of 65,335 ± 20,920 dots per area (DPA). This was followed by the TR scanner, with an average of 20,251 ± 4103 DPA. Both extraoral scanners, SC and TS, showed the numbers of triangulation points on the top surface, 15,288 ± 2717 DPA, and 14,794 ± 705 DPA, respectively (Table 2). The CO intraoral scanner recorded the least number of triangulation points, 2395 ± 1163 DPA. There was a statistically significant difference in the DPA among different scanners (*p* ≤ 0.001). Tukey’s post-hoc test showed significant differences in all paired groups, except for the TR, TS, and SC groups (Figure 6).

When comparing the reproducibility of the surface topography by the different anatomical structures, Univariate Linear Regression Analysis yielded no statistically significant differences except the type of the scanners (Table 3).

**Figure 7:**
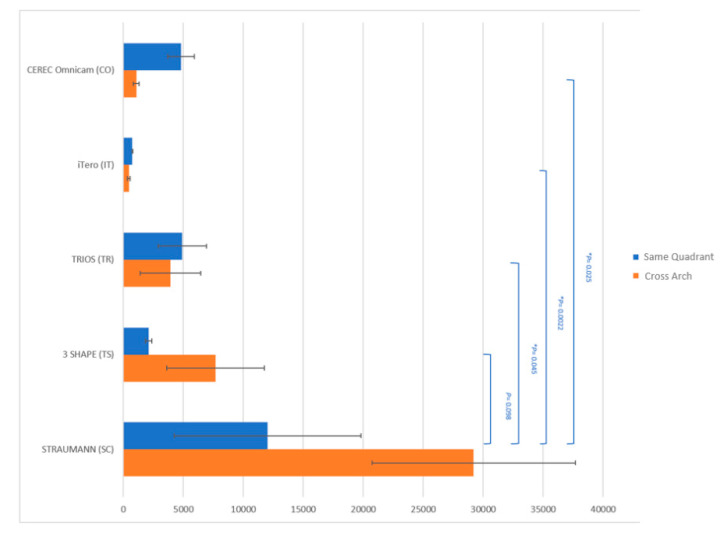
Precision of distance measurements using different scanners (same quadrant vs. cross arch). Inverse of the square of variance (1/σ^2^).

The precision of the distance measurements in the same quadrant and the cross inter-arch was calculated by an “inverse of the square of variance (1/σ^2^)”. The precision measurement value in the same quadrant between the #10 and #12 scanbodies demonstrated less evident distinctions between the extraoral and intraoral scanner groups. The SC scanner showed the highest precision measurement value of 12,026, followed by the two intraoral scanners: TR (4910) and CO (4805). Extraoral scanner TS and intraoral scanner IT produced values of 2107 and 749, respectively (Figure 7). The ANOVA test showed no statistical differences (*p* = 0.47) among the different scanners for the linear measurements in the same quadrant. A Tukey’s post hoc test revealed that the statistical difference did not exist between extraoral and intraoral scanners. 

The cross-arch precision measurement between scanbodies #5 and #12 demonstrated that extraoral scanners had significantly higher values than those of intraoral scanners. The SC scanner showed statistically significant higher precision measurements when compared to the rest of the scanners, with a value of 29,196 ± 8467; the TS scannerfollowed with a value of 7687 ± 4068. Intraoral scanners demonstrated the precision measurements in the following orders: TR (3919 ± 2509), CO (1069 ± 241), and IT (453 ± 111). The ANOVA test showed statistical differences (*p* = 0.018) among the different scanners. A Tukey’s post hoc test revealed that the statistical difference existed between SC and TR, IT, CO, respectively (Figure 7). 

## 4. Discussion

One of the requirements of an optical scanner is to create a reproducible surface topography for its use of the data. Dental scanners necessitate the detailed surface reproduction of the small-scale object in order to fabricate a precise dental prosthesis. The triangulation method is used to investigate the microtopographic inspection of surfaces. The triangulation is created on selecting the nearest three locations with measured data by forming a triangle [19]. It is designed to remove possible discontinuities between adjacent points by fitting a plane through the formed triangle. In this study, the surface topography generated from different dental scanners was evaluated by the triangulation points on the different anatomical structures and the precision of the linear measurements was studied.

The more triangulation points yield the detailed geometry of the surface. The IT scanner had the greatest number of the triangulation points on the surface of the implant scanbody compared to other scanners. However, there is no difference in reproducing the surface topography between the intraoral and extraoral scanners, except IT and CO. Unlike other scanners, software settings in IT scanner use certain areas to stitch the scan images, resulting in denser triangulation points around the selected scanbody area. Despite higher triangulation points, the IT scanner did not perform better when measuring the linear precision measurement. This critical finding suggests that having a higher number of triangulation points does not positively correlate to precision. Renne’s study demonstrated a sextant scan and complete-arch scan times for IT were 2:30 and 6:04 and for CO were 0:15 and 0:47, respectively [20]. This is of particular importance since the number of triangulation DPA may correlate directly to the scan time, but may also be independent of precision. Instead, precision is dependent upon several other factors, including: active triangulation principles, tessellations, the software algorithm, image stitching technique, and the number of scanning sensors.

To date, a number of studies have compared accuracy and precision between intraoral and extraoral scanners and have provided rather inconsistent results, reporting that extraoral scanners provide almost similar or slightly better precision [9,16,21,22,23,24]. Flugge conducted an interesting study that compared the TS extraoral scanner to the IT intraoral scanner. The study also compared scans from the same IT scanner in two different conditions: one from a direct intraoral condition and the other one from scanning extraoral models [9]. The TS scanner provided the highest precision, but surprisingly, the extraoral model scanning of the IT scanner provided higher precision. Flugge’s results suggested that intraoral conditions, such as patient movement, limited intraoral space, intraoral humidity, and saliva flow, contributed to the lower precision of a scan. Amongst inconsistent results from different studies, it is still evident that extraoral scanners provide slightly better or the same precision.

In this study, it was an interesting finding that all the scanners could generate the different anatomical structures with no statistical differences when the univariate linear regression analysis focused on determining the relationship of the parameters. The null hypothesis of comparison between the surface topography and precision was verified that it did not show the statistical differences in the relationship of anterior vs. posterior teeth, mesial vs. distal interproximal, or flat vs. contoured surface. The scanners have the capacity to generate reproducible surface topography regardless of the position and contour of the object. The discrepancy may come from the intraoral conditions of the patient which influence the accessibility of the scanners during the scanning phase. The importance of its accessibility was confirmed by a recent study which showed that partial coverage preparation and the presence of adjacent teeth resulted in significantly less accuracy due to the limitation of scan angulation and direction [25].

The null hypothesis of the precision compared from different dental scanners was rejected. The accuracy of the digital impression has so far been measured in terms of trueness, and mostly in studies with linear distance measurements [26,27,28,29]. In the linear measurement in the same quadrant of the scanbodies, the distance precision measurements indicated that the SC scanner demonstrated the highest precision, followed by the TR, CO, TS, and IT scanners. Our study results indicate that the SC scanner demonstrated the highest precision with repeated digital scans, the SC scanner provided the most consistent values as compared to the other scanning devices evaluated, however, it failed to demonstrate a statistically significant difference in precision in the same quadrant arch when compared to other scanners.

As the scope of digital dentistry expands, full-arch restorations fabricated from digital scanning are becoming more and more common. A study has shown that the trueness and precision of the full-arch digital impression are less accurate than those of a conventional impression, due to the overlapping of partial scans of both quadrants [26]. Although there was no pattern in precision measurements amongst intraoral and extraoral digital scanners, extraoral scanners were significantly more precise than intraoral scanners in the cross-arch scan. Our results in the linear cross-arch measurement follow the same pattern of another study, where the extraoral scanners (SC and TS scanners) demonstrated better precision and were statistically different from other intraoral scanners in the cross-arch scan. As mentioned previously, the SC scanner’s movable plate compensates and improves the scanning angle, thus providing significantly better precision. Most intraoral scanners use image-stitching methods to create virtual models. Such methods are prone to deviation pattern propagation, especially when scanning the anterior teeth with little geometric structure, or canine teeth at the corners of the arch which serve as transition points from the anterior to posterior teeth. On the other hand, extraoral scanners offer multiple cameras and, more importantly, multi-axis motion movements, allowing for more precise full/cross-arch scans.

Despite modeling several variables, this study did not replicate an actual clinical situation and therefore has several limitations. The master reference model was based on relatively ideal shapes of teeth without misalignment, crowding of teeth, or a severe undercut area. In addition, by scanning a gypsum model, patients’ existing restorations with different materials and their effects on digital scanning were not taken into consideration. It is important to note that although the geometry of the scanbody allowed for the measurement of two- and three-dimensional areas, the actual scanbody shape does not represent three-dimensional shapes that are found in intraoral conditions. Lastly, this study failed to account for saliva, humidity in the oral environment, patient cooperation, and operator experience. The use of digital scanners is steadily increasing: enhanced user familiarity and workflow optimization may help clinicians choose the appropriate digital scanner model after evaluating specific situations.

## 5. Conclusions

Within the limitations of the present study, the following conclusions were drawn:

1. Scanning resolution, in terms of the number of triangulation points, does not correlate to the precision. Rather, precision correlates with scanning mechanism.
2. Reproducing the surface topography did not depend on the anatomical tooth structure and position.
3. In the same quadrant, the precision in the linear measurements had no statistical difference amongst the extraoral and intraoral scanners, though the SC extraoral scanner demonstrated the highest precision.

## Figures and Tables

**Table 1:** Summary of characteristics for the scanning systems.

Scanner	Cerec Omnica (CO)	Itero Hd2.9 (IT)	Trios 3 Basic(TR)	3Shape D700 (TS)	Straumann Cares 3 Series (SC)
**Type**	Intraoral	Intraoral	Intraoral	Extraoral	Extraoral
**Manufacturer**	Sirona	Align Tech	3Shape	3Shape	Straumann
**Light Source**	White LED	Red Laser	Laser	Red Laser	Laser
**Characteristics**	Video capturing	Still image capturing	Video capturing	2 cameras, adaptive scanning tech	Model on a movable plate

**Table 2:** Summary of the number of triangulation points on the scanbody.

Scanner	CEREC OMNICAM (CO)	ITERO (IT)	TRIOS (TR)	3SHAPE D700 (TS)	STRAUMANN CARES 3 Series (SC)
Number of Triangulation on the scanbody (DPA)	2395 ± 1163.5	65,355 ± 20,920.4	20,251 ± 4103.6	14,794 ± 705.4	15,288 ± 2717.5

**Table 3:** Parameters that affect the surface topography of the scanner by Univariate Linear Regression Analysis (*p* < 0.05).

Comparison	B Coefficient
Ant. vs. Post	−0.001 (*p* = 0.985)
Flat vs. Contour	−0.066 (*p* = 0.256)
Interproximal vs. non-interproximal	0.023 (*p* = 0.713)
